# Thermal, chemical and physical analysis of VDW.1Seal, Fill Root ST, and ADseal root canal sealers

**DOI:** 10.1038/s41598-023-41798-8

**Published:** 2023-09-08

**Authors:** Shehabeldin Saber, Manar M. Galal, Amira Galal Ismail, Tamer M. Hamdy

**Affiliations:** 1 Endodontic Department, Faculty of Dentistry, The British University in Egypt, 81-11-11 El-Rehab, Cairo, 11841 Egypt; 2 Center for Innovative Dental Sciences, The British University in Egypt, El Sherouk City, Egypt; 3 Endodontic Department, Faculty of Dentistry, Ain Shams University, Cairo, Egypt; 4 Restorative and Dental Materials Department, Oral and Dental Research Institute, National Research Centre (NRC), Giza, Dokki 12622 Egypt

**Keywords:** Dental materials, Endodontics

## Abstract

This study aimed to evaluate the thermal, chemical, and physical properties of VDW.1Seal, Fill Root ST, and ADseal sealers. Thermal properties were analyzed using Thermogravimetric analysis (TGA) and Differential thermal analysis (DTA). Attenuated total reflection Fourier Transform Infrared Spectroscopy (ATR-FTIR) analysis was performed as a complementary test to confirm TGA/DTA analysis. The chemical composition of the set sealer material was identified using an X-ray powder diffraction (XRD) system. Other physical properties of each sealer were investigated; ten specimens were used to measure the solubility (at 24 h and 28 days), and another ten specimens were used to assess pH changes and calcium ion release (after 7 and 14 days). Film thickness was done according to ISO 6876 specs. The data were analyzed using the two-way ANOVA test. Results showed that for all sealers, TGA analysis revealed a direct relationship between sealer mass loss and temperature rise. In addition, the decomposition of the tested sealers started at 145 °C, 135 °C and 91 °C for VDW.1Seal, ADseal sealer, and Fill Root ST, respectively. XRD analysis revealed a higher degree of crystallinity for VDW.1Seal and ADseal. ADseal showed the least solubility; VDW.1Seal exhibited the highest alkalinity, calcium ion release, and the lowest film thickness.

## Introduction

Obturation techniques using thermo-plasticized gutta-percha are expected to fill root canal systems better than cold gutta-percha points^1^. Nevertheless, sealers are still essential to fill the gaps and voids between the core material and the root canal walls^2^. Without a sealer, both warm and cold obturation techniques will have compromised treatment outcomes^3,4^. Hydraulic calcium silicate-based sealers (HCSBS) have been recently developed as a substitute for epoxy resin-based sealers^5^. According to the source of hydration required for their setting, they are either available as powder, liquid, or premixed ready-to-use syringes^6^. In the former, hydration is initiated before insertion into the root canal. While in the latter, the residual moisture inside the root canal, along with dentine humidity, purportedly provides the water necessary for the hydration of the material^7^. Clinical studies have reported successful application of premixed HCSBS in root canal obturation^8,9^. Besides technique simplicity, HCSBS also showed favorable properties, including bioactivity^10^, biocompatibility^11^, and antibacterial potential^12^. Most are founded on the solubility of their setting reaction by-products^13^. The main component of HCSBS is tricalcium silicate, which, when hydrated, forms a calcium silicate hydrate matrix and calcium hydroxide that leach out to interact with the surrounding environment^14,15^. Recently, new premixed sealers were marketed with innovative formulations to upgrade their clinical performance. In comparison to previous HCSBS, VDW.1Seal (VDW, München, Germany) contains relatively less tricalcium silicate (5–15 wt %) and more zirconium dioxide (50–70 wt %) in addition to dimethyl sulfoxide as filler. Fill Root ST (Dental World, Molfetta, Italy) is another recently introduced premixed sealer based on calcium aluminosilicate and zirconium dioxide. Currently, there is scarce data about their physico-chemical properties in terms of solubility, alkalinity, calcium ion release, and film thickness. Likewise, it is necessary to investigate their thermal stability to determine if they can tolerate the heating temperatures generated during thermal-based obturation techniques without decomposition. Such data would give clinicians insights for better selection of the sealer type to be compatible with heat application during obturation. Therefore, this in vitro study aimed to investigate the thermal stability and some physico-chemical properties (short- and long-term solubility, pH changes, calcium ions release, and film thickness) of VDW.1Seal and Fill Root ST in comparison to an epoxy resin-based sealer, ADseal Root canal sealer (Meta BioMed, Cheongju, Korea). The null hypothesis was that there were no differences between the tested materials.

## Materials and methods

All methods were carried out following relevant guidelines and regulations. The research ethics committee at the Faculty of Dentistry at the British University in Egypt approved all the experimental protocols. Since the data were evaluated retrospectively, pseudonymously, and were solely obtained for treatment purposes, a requirement of informed consent was waived by the ethics committee at the Faculty of Dentistry, The British University in Egypt (approval number 22-031, date 18/12/2022).

The root canal sealers used in the current study are represented in Table 1.

**Table 1:** Materials used in this study.

Materials	Manufacturers	Composition
ADseal (epoxy resin-based root canal sealer)	*Metabiomed, Cheongju, Korea*	Poly(1,4-butanediol) bis(4-aminobenzoate), Bisphenol A diglycidyl ether-bisphenol A copolymer, 2-Hydroxyethyl salicylate, Triethanolamine, Calcium oxide
VDW.1Seal (Hydraulic calcium silicate-based sealer)	*VDW, München, Germany*	Zirconium Dioxide, Tricalcium silicate, Dimethyl sulfoxide, Lithium carbonate
Fill Root ST (Hydraulic calcium silicate-based sealer)	*DW Dental World. Molfetta (BA)—Italy*	Calcium aluminosilicate, zirconium dioxide

### TGA/DTA analysis

Approximately 16 mg of powder for each set of sealers was obtained by grinding using an agate mortar and pestle (Nahita, Istanbul, Turkey). Sample powders were analyzed to evaluate the thermal stability of each sealer using Thermogravimetric analysis (TGA) and Differential thermal analysis (DTA) using a TGA/DTA Thermal Analyzer (Shimadzu DTG-60H Thermal Analyzer, Kyoto, Japan). Thermal measurements were performed under the flow of nitrogen atmosphere with a flow rate of 100 ml min^−1^ in the temperature range from ambient to 250 °C. The heating rate was 20 °C per minute. Highly sintered α-Al_2_O_3_ was used as the reference material. Thermo-analytical TGA and DTA curves were obtained simultaneously. The data were analyzed using the TGA software (CDSS, v1.1).

### ATR-FTIR test

Attenuated total reflection Fourier Transform Infrared Spectroscopy (ATR-FTIR) analysis (Nicolet 6700 FTIR instruments, Thermo Fisher Scientific, Waltham, USA) was performed on the set sealers as a complementary test to confirm thermal stability results. FTIR analysis was performed in the spectral range (4000–500 cm^1^) following the previously described procedure^16^.

### XRD analysis

The chemical composition of the set sealer material was investigated using an X-ray powder diffraction (XRD) system (Bruker-AXS D8 X-ray diffractometer, Germany) to identify the existing crystalline phases and measure the degree of crystallinity. The crystalline structure of the test sealer was determined by passing an X-ray beam of a known wavelength into the specimen while rotating it at an angle. The intensity of X-rays from the sample was measured by a detector^14^, while the crystallinity was evaluated by careful evaluation of the baseline to peak separation in an extended scan range using DIFFRAC.EVA software (Bruker AXS GmbH). XRD data were collected in the 2θ range 0–60° under 30.0 mA, 40.0 kV, and a scan rate of 4°/min. The obtained XRD patterns were characterized using the Joint Committee on Powder Diffraction Standard (JCPDS) databases.

### Analysis of the physical properties

#### Sample size calculation

Power calculation was performed using G*Power 3.1.9.7 (Heinrich Heine University, Dusseldorf, Germany). Based on the results of previous studies^17–19^ and using an alpha (α) level of 0.05 (5%) and a Beta (β) level of 0.20 (20%), i.e., power = 80%, the predicted minimum sample size (n) was nine specimens in each group. Thus, ten samples were prepared per group for evaluation of solubility, and another ten samples were prepared per group for evaluation of pH change and calcium ion release. Concerning film thickness testing, three tests were carried out according to ISO 6876 specs.

### Specimen preparation

Root canal sealers were carefully injected into the specially designed molds for each test type.

### Solubility

Solubility was determined according to the standards set by the International Standard Organization (ISO 6876:2012)^20^. Short- and long-term solubility were determined after 24 h and 28 days, respectively, following the previously described procedures^20,21^. Cylindrical polyethylene molds with dimensions of 1.5 mm height and 7.75 mm inner diameter were used and filled with each root canal sealer to acquire disc-shaped specimens (n = 10 per sealer), which were incubated at 37 °C and 95% relative humidity for 24 h to set. After setting, the specimens were removed from the molds and weighed three times to determine the mean mass of each specimen (M1) using a microbalance. Then, all set sealer specimens were stored separately in plastic flasks containing 7.5 mL of distilled water in an incubator at 37 °C and 95% relative humidity. The specimens were removed from the incubator, bench dried, and re-weighed (M2) after 24 h and 28 days of storage. Mass loss was expressed as a percentage of the original mass^20,21^. The percentage of root canal sealer solubility was calculated as follows: (M1-M2)/M1 X 100%^20^.

### pH change

Cylindrical polytetrafluoroethylene tubes were used to shape each type of root canal sealer into disc specimens (n = 10) with a 5 mm diameter and 2 mm thickness. After the sealer setting, each specimen was soaked in 10 mL of distilled water and incubated at 37 °C and 95% relative humidity for 7 and 14 days. Then the pH change of the solution was measured immediately and after 7 and 14 days of immersion by using an electrode pH meter (Jen-way 3510 bench pH meter, UK)^20^. The accuracy of the pH meter was auto calibrated with calibration solutions (pH 4, 7, and 10; Merck, Darmstadt, Germany) every 48 h. After each reading, the electrode was flushed with distilled water and dried before taking another reading. The temperature of the room during measurements was adjusted to 25 °C.

### Calcium ions release

The previous solutions were used to measure the release of calcium ions using inductively coupled plasma optical emission spectroscopy (ICP-OES) (Ultima 2 ICP, Horiba, USA). The cumulative amounts of the degradable calcium ions released from each sealer were measured after 7 and 14 days, respectively (mg/L)^20^.

### Film thickness

Assessment of film thickness was evaluated according to the International Standard Organization (ISO) 6876/2012 instructions^20^. Two flat glass plates (5 mm in thickness, 200 ± 10 mm^2^ surface area) were placed over one another. Their total thickness was measured by a digital micrometer to the nearest 10 μm. Then 0.5 ml of each sealer was dispensed onto the first plate, and the second plate was placed over it. A 150 Newton load was vertically applied for 3 min on the upper glass plate. The total thickness of the plates, including the sealer, was re-measured 7 min after the time of load application. The total thickness of the plates was deducted from this amount, and the film thickness of the sealer was obtained. The mean value of the film thickness for each sealer was recorded by repeating the test three times and calculating the average value^20^.

### Statistical analysis

The results were collected and analyzed statistically using a two-way ANOVA. Tukey’s post-hoc test was conducted for multiple comparisons. The significance level was set at (P ≤ 0.05). Data were managed using SPSS 16.0 statistical software (SPSS Inc., Chicago, IL, USA).

## Results

### Thermal and chemical analysis results

**Figure 1:**
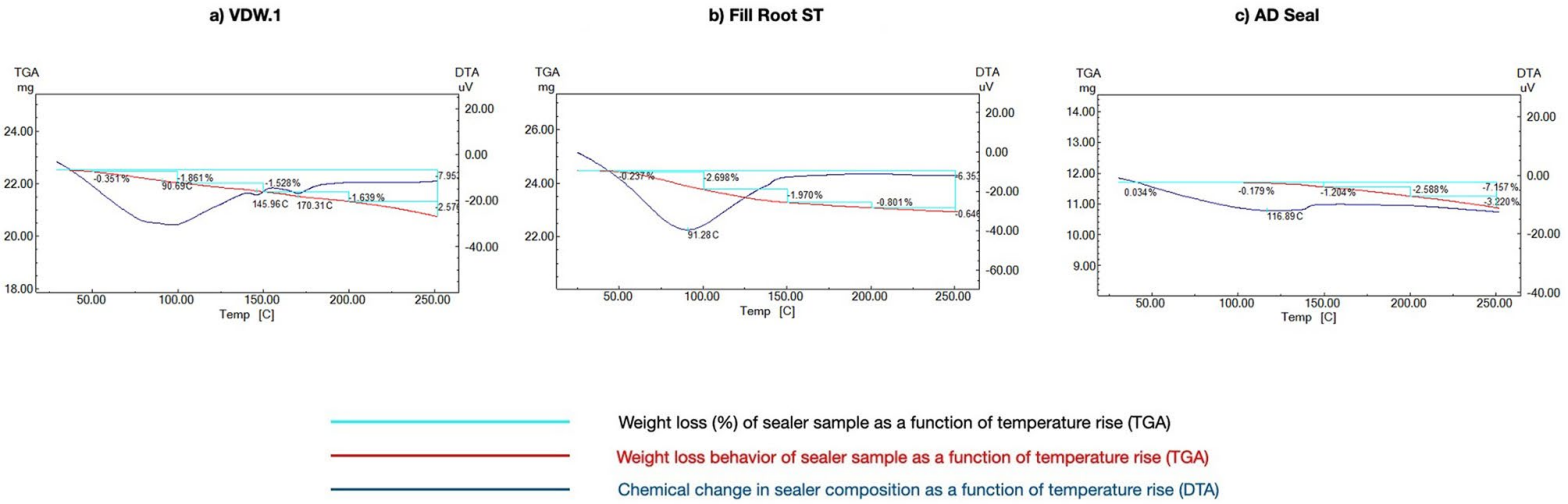
DTA/TGA analysis of all tested sealers. (**a**) VDW.1Seal root canal sealer. (**b**) Fill Root canal ST sealer. (**c**) ADseal Root canal sealer).

DTA/TGA results for tested sealers are represented in Fig. 1a–c. The blue-colored curve represents the chemical change in the sealer’s chemical composition (Differential Thermal Analysis, DTA). The red-colored curve shows the weight loss behavior of the sealer sample as a function of increasing the temperature (Thermal Gravimetric Analysis, TGA). The curve in turquoise gives the percentage (%) of weight loss as a function of the increasing temperature (Thermal Gravimetric Analysis, TGA).

TGA/DTA data is represented in Fig. 1. DTA data of VDW.1Seal (Fig. 1a) showed a medium, very broad endothermic band around 90 °C and two very weak two endothermic peaks at 145 °C and 170 °C, respectively. TGA data (Table 2) revealed a total weight loss of 7.9%. DTA data of Fill Root ST canal sealer (Fig. 1b) showed a strong, broad endothermic band around 91°C with a total weight loss of 2.93% starting from 60 °C and ending at 91°C as indicated from TGA results (Table 2). DTA data of ADseal Root canal sealer (Fig. 1c) showed a weak, very broad band in the range 50–135 °C with a total weight loss of about 7.2%, as revealed from TGA data (Table 2).

**Table 2:** TGA analysis of the examined sealers.

Sealer	ADseal	VDW.1Seal	Fill Root ST	P value
Solubility (short-term) after 24 h	1^aI^ ± 0.16	2.18^bI^ ± 0.08	3.32^cI^ ± 0.13	P = 0.0001*
Solubility (long-Term) after 28 days	1.24^aII^ ± 0.11	2.82^bII^ ± 0.08	4.76^cII^ ± 0.11	P = 0.0001*
P value	P = 0.02*	P = 0.0001*	P = 0.0001*	

**Figure 2:**
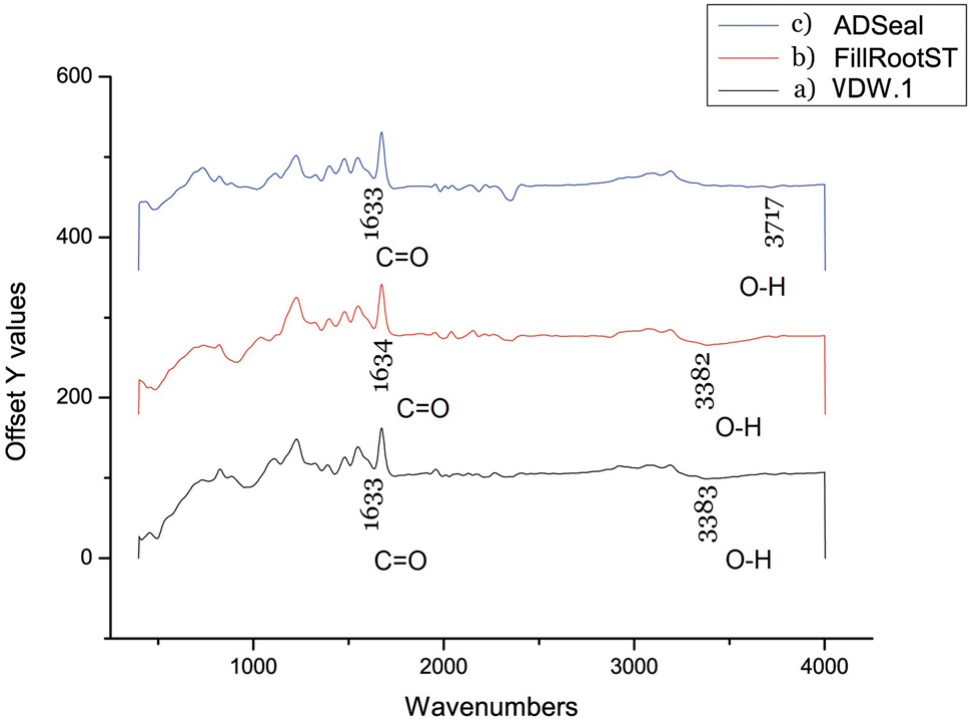
FTIR analysis of all tested sealers. (**a**) VDW.1Seal root canal sealer. (**b**) Fill Root canal ST sealer. (**c**) ADseal Root canal sealer).

The ATR-FTIR analysis of the tested sealers is represented in Fig. 2. The VDW.1Seal sample revealed a very weak broad band at 3383 cm^−1^ and a medium-strong band at 1633 cm^−1^. While ATR-FTIR analysis of the Fill Root ST canal sealer sample revealed a very weak broad band at 3382 cm^−1^ and a medium-strong band at 1634 cm^−1^, Moreover, ATR-FTIR analysis of the ADseal root canal sealer sample revealed a very weak broad band at 3717 cm^−1^ and a medium-strong band at 1633 cm^−1^.

**Figure 3:**
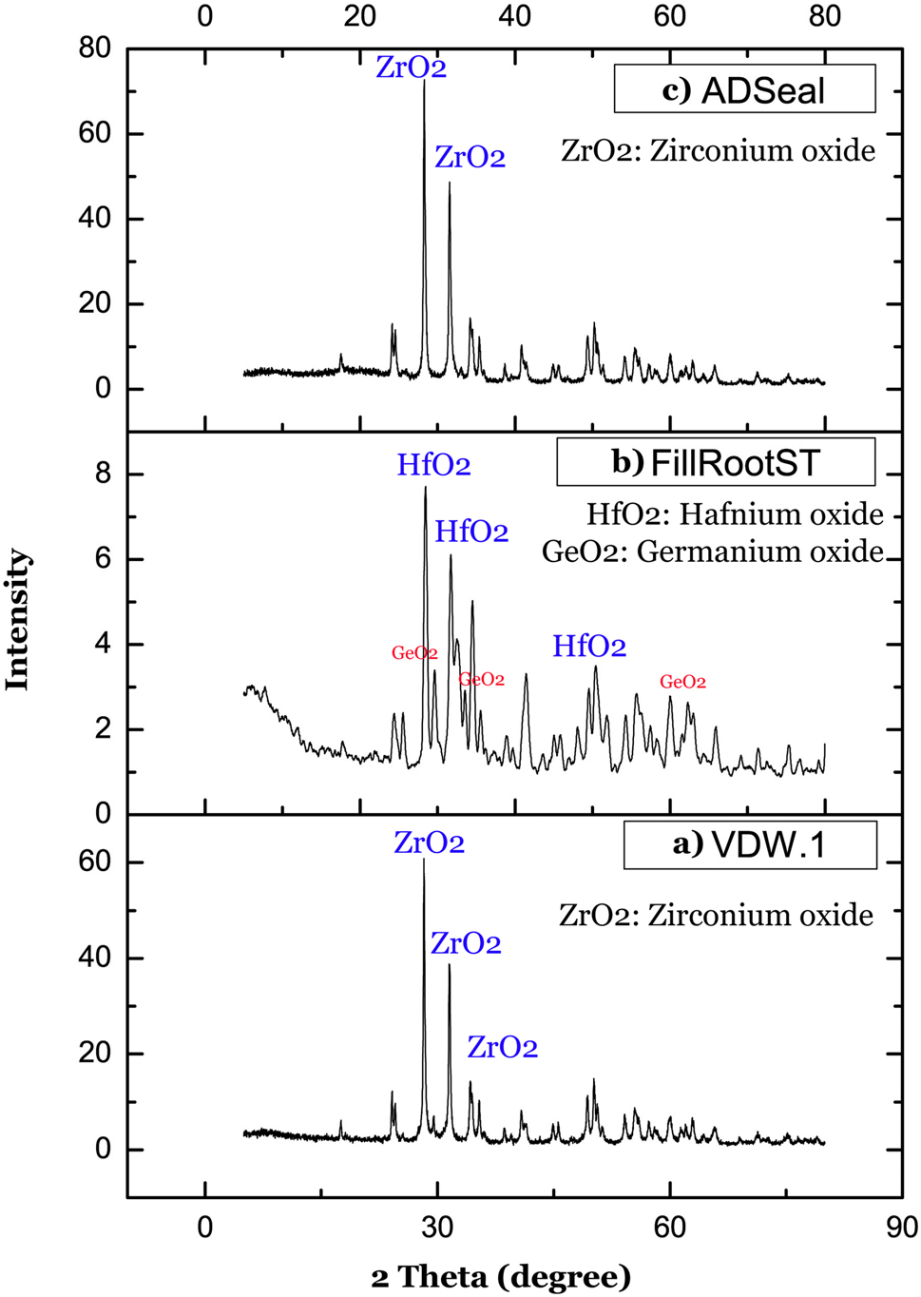
XRD analysis of all tested sealers. (**a**) VDW.1Seal root canal sealer. (**b**) Fill Root canal ST sealer. (**c**) ADseal Root canal sealer).

The XRD analysis of VDW.1Seal (Fig. 3a) revealed that the degree of crystallinity is about 73%, with crystalline diffraction peaks at 2θ values of 28.6°, 31.5°, and 44.3° that exhibited peaks for ZrO_2_ (ICDD File No. 37-1484). The XRD analysis of Fill Root canal sealer (Fig. 3b) revealed that the degree of crystallinity is about 67%, with crystalline diffraction peaks at 2θ values of 28.4°, 31.7°, and 49.5° that exhibited peaks for Hafnium oxide (ICDD File No. 43-1017), and values of 33.5°, 34.5°, and 60.0° that exhibited peaks for Germanium oxide (ICDD File No. 65-0333). The XRD analysis of ADseal root canal sealer (Fig. 3c) revealed that the degree of crystallinity is about 74%, with crystalline diffraction peaks at 2θ values of 28.29°and 31.5° that exhibited peaks for ZrO_2_ (ICDD File No. 37-1484).

### Physical analysis results

#### Solubility

Solubility results are reported in Table 3. There was a significant difference in the solubility between the three types of sealers after 24 h and after 28 days (*p* = 0.0001*). The ADseal showed the least solubility (1 and 1.24%), while the Fill Root ST presented the highest solubility (3.32 and 4.76%). Within the same material, there was a significant increase in solubility with time (*p* ≤ 0.05).

**Table 3:** Solubility % of the different sealers at different time intervals.

	ADseal (%)	VDW.1Seal (%)	Fill Root ST (%)
Mass loss at 50 °C	0.034	0.35	0.23
Mass loss at 100 °C	0.213	2.21	2.93
Mass loss at 150 °C	1.41	3.74	4.90
Mass loss at 200 °C	4.0	5.37	5.71
Mass loss at 250 °C	7.22	7.95	6.35

Means with different small letters in the same row indicate statistically significance difference, while means with different capital roman letters in the same column indicate statistically significance difference. *Corresponds to statistically significant difference (P ≤ 0.05).

### pH changes results

The results are reported in Table 4. The pH recorded for all sealers increased over time, which was significant for the VDW.1Seal and Fill Root ST sealers (*p* < 0.05). At all observation points, the highest pH value was recorded for the VDW.1Seal sealer and the lowest was recorded for the ADseal sealer (*p* < 0.05).

**Table 4:** The pH values of the different sealers at different time intervals.

Sealer	ADseal	VDW.1Seal	Fill Root ST	P value
Immediate	9.39^aI^ ± 0.13	9.88^bI^ ± 0.17	9.72^bI^ ± 0.16	P = 0.001*
7 days	9.62^aII^ ± 0.15	11.46^cII^ ± 0.24	10.57^bII^ ± 0.1	P = 0.001*
14 days	10.29^aIII^ ± 0.11	12.39^cIII^ ± 0.15	11.48^bIII^ ± 0.11	P = 0.001*
P value	P ≤ 0.04	P = 0.0001*	P = 0.0001*	

Means with different small letters in the same row indicate statistically significance difference, while means with different capital roman letters in the same column indicate statistically significance difference. *Corresponds to statistically significant difference (P ≤ 0.05).

### Calcium ions release results

The results are reported in Table 5. The calcium ion release recorded for all sealers increased over time, which was significant for all sealers (*p* < 0.05). While for both observation points, the highest release was recorded for the VDW.1Seal sealer (17.86 and 48.62 mg/l) and the lowest for the ADseal sealer (0.22 and 1.5 mg/l) (*p* < 0.05).

**Table 5:** Calcium ions release (mg/l) of the different sealers at different time intervals.

Sealer	ADseal	VDW.1Seal	Fill Root ST	P value
7 days	0.22^aI^ ± 0.08	17.86^cI^ ± 1.44	10.44^bI^ ± 0.55	P = 0.0001*
14 days	1.05^aII^ ± 0.16	48.62^cII^ ± 1.31	26.84^bII^ ± 1.21	P = 0.0001*
	P = 0.0001*	P = 0.0001*	P = 0.0001*	

Means with different letters in the same row indicate statistically significance difference, while means with different capital roman letters in the same column indicate statistically significance difference. *Corresponds to statistically significant difference (P ≤ 0.05).

### Film thickness results

The VDW.1Seal showed the lowest film thickness of (49.4 ± 2.07) µm, followed by the ADseal sealer (80.6 ± 1.14) µm, while the thickest was the Fill Root ST sealer (90 ± 4.3) µm, and these differences were statistically significant (*p* < 0.001).

## Discussion

Improvement in root canal shaping instruments and obturation materials is required to achieve better treatment outcomes^21^. The current in vitro study sought to assess a series of physicochemical properties of the newly introduced HCSBS (VDW.1 Seal and Fill Root ST) in comparison to an epoxy resin-based sealer (ADseal Root canal sealer), which was used instead of AH plus, and was less extensively investigated despite having more favorable biological properties^11^. Thermogravimetric analysis (TGA) and Differential thermal analysis (DTA) were chosen to investigate whether the tested sealers can withstand the temperatures generated inside the root canals during thermal-based obturation techniques without decomposition and, therefore, allow clinicians to predict their clinical behavior when subjected to heat and achieve better clinical decisions for higher treatment outcomes.

TGA measures the weight loss over a temperature range, while DTA determines the endothermic and exothermic event temperatures of the materials at the onset of melting and crystallization. The thermal analysis determines the behavior and thermal stability of the materials upon heating^22–24^. The chemical structure of the sealer and the predicted behavior could be complementary assessed by using ATR-FTIR analysis, which is an accurate and quick analytical method that requires minimal samples^25,26^. Moreover, non-destructive XRD analysis was done to detect the degree of crystallinity and identify the crystallographic phases of the tested materials^27^.

Most commercial thermal obturation devices have four temperature settings (150 °C, 180 °C, 200 °C, and 230 °C). Although it has been shown that the actual temperature generated inside the root canal is less than that on the device display^2^, for each TGA curve, we opted to investigate the sealer mass loss at 50 °C, 100 °C, 150 °C, 200 °C, and 250 °C to assess the thermal stability of the tested sealers before, during, and after this temperature range.

TGA/DTA thermal analysis for all tested sealers revealed a broad endothermic band below 100 °C. These losses may be attributed to physical adsorbed moisture (H_2_O). To further explore this finding, ATR-FTIR was conducted at ambient temperature, and it revealed stretching and scissors bands of 3383.92, 3382.45, and 3717.21 for VDW.1Seal, Fill Root ST, and ADseal, respectively. This is suggestive of the release of moisture (the OH of water). Moreover, the other peak bands of 1633, 1634, and 1633 were recorded at higher temperatures for VDW.1Seal, Fill Root ST, and ADseal, respectively, which may be due to stretching and scissors bands of free H_2_O.

For all sealers, TGA/DTA analysis revealed a direct relationship between sealer mass loss and temperature rise. The decomposition of the tested sealers started at 145 °C, 135 °C and 91 °C for VDW.1Seal, ADseal sealer, and Fill Root ST, respectively. This can be primarily explained based on the XRD analysis that revealed a higher degree of crystallinity (73% and 74%) for VDW.1Seal and ADseal, respectively, as well as their inclusion of the stable zirconium oxide ceramic fillers, which resist thermal decomposition up to 2000 ºC^28,29^, in comparison with the Fill Root ST whose crystallinity was only 67%.

Similar favorable thermal stability of HCSBS was reported by Donnermeyer et al. 2021^30^ and 2022^5^, Vipiana et al. 2015^2^, and Atmeh and AlShawaimi 2017^31^, who also reported that heating of epoxy resin sealers resulted in time-dependent irreversible structural changes, though associated with less mass loss (1.2% at 250 ^o^C). In contrast, Donnermeyer et al. 2020^32^ showed that heating an epoxy resin sealer up to 97 °C for 60 s did not lead to any substantial physical or chemical changes. It is worth mentioning that the actual temperatures delivered by heat pluggers are much lower than the temperatures shown on the device display^2,33^.

Although the crystallinity of the dental materials provides thermal stability and preservation of the structural integrity against degradation and solubility of the materials by the action of the environment^34,35^, still, the presence of some degree of amorphous phase permits a reasonable grade of solubility and ions leaching responsible for pH changes and precipitation, which are essential for remineralization, sealing, and tissue healing^36–38^.

The solubility of the tested sealers was investigated in the short term after 24 h and in the long term after 28 days. Regarding the short-term solubility, the least soluble was the ADseal sealer, owing to its resinous nature in addition to the highest degree of crystallinity of its composition, followed by the VDW.1Seal, with a comparable degree of crystallinity and the presence of the highly stable zirconium oxide fillers, while the most soluble was the Fill Root ST sealer, which exhibited the least degree of crystallinity. Regarding long-term solubility, there was a significant increase in the solubility of all sealers (*p* ≤ 0.05), which may be due to the increased interaction with the surrounding aqueous environment. It was noted that only ADseal and VDW.1Seal root canal sealers fulfilled the minimum requirements of solubility described by ISO 6876:2012, which is 3% of the initial mass by 24 h. It is worth mentioning that solubility values reported from laboratory tests may not disclose the actual clinical behavior of materials. Using water as a storage medium may overemphasize the solubility^39^, whereas physiological solutions could encourage mineral deposition and increase rather than decrease the net mass^22^, resulting in different values for the solubility of the same material when tested using different storage media^40^.

To predict their biological behavior, pH changes and calcium ion release for the tested sealers were investigated at 7 and 14 days, representing the maximum peaks for calcium ion release and a subsequent increase in pH value^19,41,42^. The alkalinity of the hydraulic calcium silicate-based sealers is regarded as one of their chief advantages, as it leads to the formation of apatite deposits on the sealer surface after contact with body fluids^43^. This interaction enhances bioactivity and promotes a strong chemical bond^44^. Moreover, the alkaline pH provides bacteriostatic effects^45^ and can promote apical healing and tissue mineralization^36^. Whereas the release of calcium ions, which are strong extracellular signals for undifferentiated cells^46^, is important for healing of apical periodontitis associated with bone resorption. Results of our study showed that VDW.1Seal exhibited the highest alkalinity and the highest cumulative calcium ion release compared to the other sealers at the end of the observation period (*p* ≤ 0.05). Despite the relatively low amount of calcium silicate (5–15%) present in its composition^47^, a high open pore volume was reported for a sealer with a similar composition that promoted higher water absorption and ion release. This does not essentially predict poor clinical performance of the sealer, as nucleation of apatite and carbonate may recompense the behavior of the sealer^48^. The significantly higher calcium and hydroxyl ion release of hydraulic calcium silicate sealers in comparison with epoxy resin-based sealers was also reported by previous studies^5,17,19,49,50^. The film thickness of the root canal sealers is essential because thin film thicknesses enhance the wettability of the materials on the canal walls, providing appropriate sealing^51^. According to the results of this study, all tested sealers fulfilled the ISO recommendation for sealer film thickness (50 μm)^5,20,52,53^. The differences between the sealers were statistically significant (*p* ≤ 0.05), with VDW.1 Seal having the lowest film thickness, mostly due to its smaller particle size.

Limitations of this study include no assessment of the amorphous phases of the sealers was performed. Moreover, the study does not monitor the chemical changes before moisture adsorption. Furthermore, it is recommended to perform a further study to investigate the chemical elemental analysis using X-ray fluorescence (XRF). In addition, it is recommended to investigate the chemical changes before, during, and after heating by FTIR, considering the time of heat application, and compare the physicochemical properties of freshly mixed and set root canal sealers, preferably with enhanced testing methodologies that can allow better clinical translation of the results.

## Conclusions

According to the findings of this study, the null hypothesis is rejected as significant differences existed among the tested sealers. It could be concluded that the chemical composition and the degree of crystallinity greatly influence the thermal stability and solubility of the root canal sealers. Moreover, the thermal stability could be increased by the incorporation of stable zirconium oxide. Furthermore, VDW.1Seal exhibited the most favorable physicochemical properties and can be compatible with thermal-based obturation techniques.

## Data Availability

The datasets generated during and/or analyzed during the current study are available from the corresponding author on reasonable request.
